# Effectiveness of digital mental health interventions for university students: an umbrella review

**DOI:** 10.7717/peerj.13111

**Published:** 2022-03-31

**Authors:** Sophia Harith, Insa Backhaus, Najihah Mohbin, Huyen Thi Ngo, Selina Khoo

**Affiliations:** 1 Centre for Sport and Exercise Sciences, Universiti Malaya, Kuala Lumpur, Malaysia; 2 Institute of Medical Sociology, Centre for Health and Society, Medical Faculty, Heinrich Heine University, Düsseldorf, Germany; 3 Health Promotion Unit, Pekan District Health Office, Pekan, Pahang, Malaysia; 4 Faculty of Library and Information Science, University of Social Sciences and Humanities/Vietnam National University, Ho Chi Minh City, Vietnam

**Keywords:** Digital intervention, Digital health, Mental health, mHealth, Young people, University students, Undergraduate

## Abstract

**Background:**

Poor mental health among university students remains a pressing public health issue. Over the past few years, digital health interventions have been developed and considered promising in increasing psychological wellbeing among university students. Therefore, this umbrella review aims to synthesize evidence on digital health interventions targeting university students and to evaluate their effectiveness.

**Methods:**

A systematic literature search was performed in April 2021 searching PubMed, Psychology and Behavioural Science Collection, Web of Science, ERIC, and Scopus for systematic reviews and meta-analyses on digital mental health interventions targeting university students. The review protocol was registered in the International Prospective Register of Systematic Reviews PROSPERO [CRD42021234773].

**Results:**

The initital literature search resulted in 806 records of which seven remained after duplicates were removed and evaluated against the inclusion criteria. Effectiveness was reported and categorized into the following six delivery types: (a) web-based, online/computer-delivered interventions (b) computer-based Cognitive Behavior Therapy (CBT), (c) mobile applications and short message service (d) virtual reality interventions (e) skills training (f) relaxation and exposure-based therapy. Results indicated web-based online/computer delivered-interventions were effective or at least partially effective at decressing depression, anxiety, stress and eating disorder symptoms. This was similar for skills-training interventions, CBT-based intervention and mobile applications. However, digital mental health interventions using virtual reality and relaxation, exposure-based therapy was inconclusive. Due to the variation in study settings and inconsistencies in reporting, effectiveness was greatly dependent on the delivery format, targeted mental health problem and targeted purpose group.

**Conclusion:**

The findings provide evidence for the beneficial effect of digital mental health interventions for university students. However, this review calls for a more systematic approach in testing and reporting the effectiveness of digital mental health interventions.

## Introduction

The prevalence of mental health problems amongst university and college students remains a pressing and urgent issue. In 2018, the World Health Organization reported that approximately one-third of first-year university students from 19 universities (13,948 respondents) across eight countries (Australia, Belgium, Germany, Mexico, Northern Ireland, South Africa, Spain, and United States) screened positive for at least one common DSM-IV anxiety, mood, or substance disorder (Auerbach et al., 2018). Similar results have been found in another international study of 12 countries in Europe (Albania, Germany, Italy, Kosovo, Switzerland), Asia (Malaysia, Oman, South Korea, Taiwan), Latin and North America (Brazil, United States), and Australia with 48% of students presenting clinically relevant depressive symptoms (Backhaus et al., 2020). Due to the recent COVID-19 pandemic, an increased prevalence of mental health problems amongst university students have been reported, which may have exacerbated this pressing issue (Hamza et al., 2021; Li et al., 2021; Wathelet et al., 2020).

For students, university years position themselves during a pivotal development transition towards emerging adulthood (Sussman & Arnett, 2014). This represents a period that encompasses the bearing of greater responsibilities and identity exploration centered within a new social context, which is not necessarily stable (Auerbach et al., 2018; Sussman & Arnett, 2014). Life as a university student can be filled with unpredictability, including constant changes to social groups and academic-related choices that ultimately can cause a greater amount of stress and reduce social support, which are contributing factors to mental health problems (Slavich & Auerbach, 2018). Karyotaki et al. (2020) found that the majority of students (93.7%) reported at least some stress in six life areas (financial situation, health, love life, relationship with family, relationship at work/school, problems experienced by loved ones).

In the US, a national survey reported that the most common problems affecting academic performance during university were stress, anxiety, and depression (American College Health Association, 2017). This can cause detrimental consequences such as termination of education, poor academic performance, subsequently reducing employment prospects in adulthood. Furthermore, other bio-psychosocial stressors such as living away from family, concerns over future employment (Pan et al., 2016), financial conditions (Richardson et al., 2017), psychological conditions such as poor resiliency, academic pressures, competitive environment, and interpersonal relationships (Waqas et al., 2015) and low levels of cognitive and behavioral social capital (Backhaus et al., 2020) were found to be associated with depressive symptoms amongst students.

Current efforts to alleviate these mental health problems include supporting on-campus facilities such as health centers equipped to provide mental health support to nurture their students’ wellbeing (Xiao et al., 2017). This may include counselling sessions and organized activities. However, concerns have been voiced about structural and psychological barriers such as understaffed facilities and timetable constraints (Xiao et al., 2017) as well as stigmatization to seek assistance, lack of motivation and discomfort of visiting a therapist (Hadler et al., 2021). These barriers may hinder students in need from seeking psychological assistance. Consequently, to increase facility capacity and to provide mental health counselling to all students in need, universities across the globe have started to implement digital mental health interventions. Digital mental health interventions have been widely acknowledged to have the potential to alleviate some of these accessibility barriers, which have been exhibited across various populations *i.e*., older adults and children (Liverpool et al., 2020; Seifert, Reinwand & Schlomann, 2019).

Digital mental health interventions are mental health support that is delivered *via* web-based or mobile-based platforms, which further denotes itself as eHealth and mHealth interventions (WHO, 2016). This includes successfully adapted web-or mobile-based delivery of evidence-based psychotherapies such as cognitive-behavioural trials. Studies have reported that digital mental health interventions can effectively treat depression, anxiety, sleep, stress, alcohol use disorders, post-traumatic stress disorders and eating disorders amongst college students (Hadler et al., 2021). Young people are considered one of the most connected groups due to their exposure to digital communications (Winther, Livingstone & Saeed, 2019). They have also reported a preference for the internet as a source of seeking health-related information to address or solve personal health problems and concerns (Burns et al., 2010; Stellefson et al., 2011).

The effectiveness of digital mental health interventions among university students is a growing field of research, with more publications focusing on the efficacy for improving symptoms of various mental health problems and enhancing psychological wellbeing, being published each year. Thus, the main objective of this umbrella review is to critically evaluate, synthesize, and summarize available reviews and meta-analyses investigating the effectiveness of various digital mental health interventions amongst university students.

## Materials and Methods

This umbrella review was conducted in accordance with the Preferred Reporting Items for Systematic Reviews and Meta-Analyses (PRISMA) (Page et al., 2021), which helps review authors to improve the reporting of their systematic review and/or meta-analysis and guidelines developed by Aromataris et al. (2020), which outline how to conduct an umbrella review. A detailed protocol for the review has been registered with the International Prospective Register of Systematic Reviews PROSPERO [CRD42021234773].

### Search strategy and selection criteria

The included reviews were identified through searching the following online databases: PubMed, Psychology and Behavioural Science Collection, Web of Science, ERIC, and Scopus. The publication date was limited from January 1, 2000, till February 26, 2021, due to the increased use of digital interventions amongst developed and developing nations which began in the early 2000s (WHO, 2016). The search strategy can be found in Supplemental Material 1. All search fields were considered regardless of the language of publication. The type of publications was limited to reviews.

Four independent reviewers (SK, HY, SH and NM) performed the search strategy. To reduce bias and subjectivity article search, article selection and data extraction was performed in duplicate and independently. More specifically, two reviewers (SK and HY) independently performed the literature search through the selected databases. Another two reviewers (SH and NM) conducted the title and abstract screening. Disagreements were resolved by a third reviewer (IB). The full text of relevant reviews was then independently evaluated by two reviewers (SH and NM) to finalize its eligibility. Disagreements between the reviewers were resolved during a consensus session with a third reviewer (IB).

### Inclusion and exclusion criteria

This umbrella review included reviews that (a) explored interventions that aimed to improve an individual’s psychological wellbeing (b) were delivered *via* a digital platform (c) specifically available for university students, irrespective of gender, ethnicity, age, or any other social demographic characteristics. Both eHealth and mHealth interventions were included. eHealth refers to the use of information and communication technologies to support health support services, typically online and offline computer-based interventions (WHO, 2016). mHealth is the use of mobile devices to support medical and public health practices (WHO, 2016). This umbrella review included systematic reviews, meta-analyses, scoping reviews, and rapid reviews. Observational studies (cohort and case-control studies), Randomized Controlled Trials (RCT), Controlled Trials (CT) studies, reviews that incorporated theoretical studies or published opinion, and non-systematic or narrative reviews were excluded. Publications were not limited by language.

### Data extraction

To minimize the risk of error and bias, the data extraction followed a two-step approach and was done by two independent reviewers (SH & NM). As a first step, a first reviewer (SH) independently extracted the data using a pre-defined extraction table, followed by a second reviewer (NM), who thoroughly checked the data entry to ensure that nothing was missed. Disagreements between the reviewers were resolved during a consensus session with a third reviewer (IB).

The following study characteristics were extracted:

- study background information,
- intervention-related information,
- outcome-related information,
- overall quality and risk of bias assessments of primary studies, and
- funding of the study.

### Quality assessment

Two reviewers (IB and SH) independently evaluated the methodological quality of the included reviews utilizing the AMSTAR-2, which is a recognized critical appraisal tool used for systematic reviews and meta-analyses on healthcare interventions (Shea et al., 2017). Results were compared and there were no disagreements in the assessment.

### Data synthesis

We narratively synthesized evidence from systematic reviews and meta-analyses due to the great heterogeneity of digital mental health interventions and effect sizes provided investigated in the systematic reviews and meta-analyses. Furthermore, to organize the amount of information provided in the systematic reviews and meta-analyses, we have categorised the interventions in two main ways; (a) type of intervention and (b) technology delivery method.

## Results

### Search results

**Figure 1:**
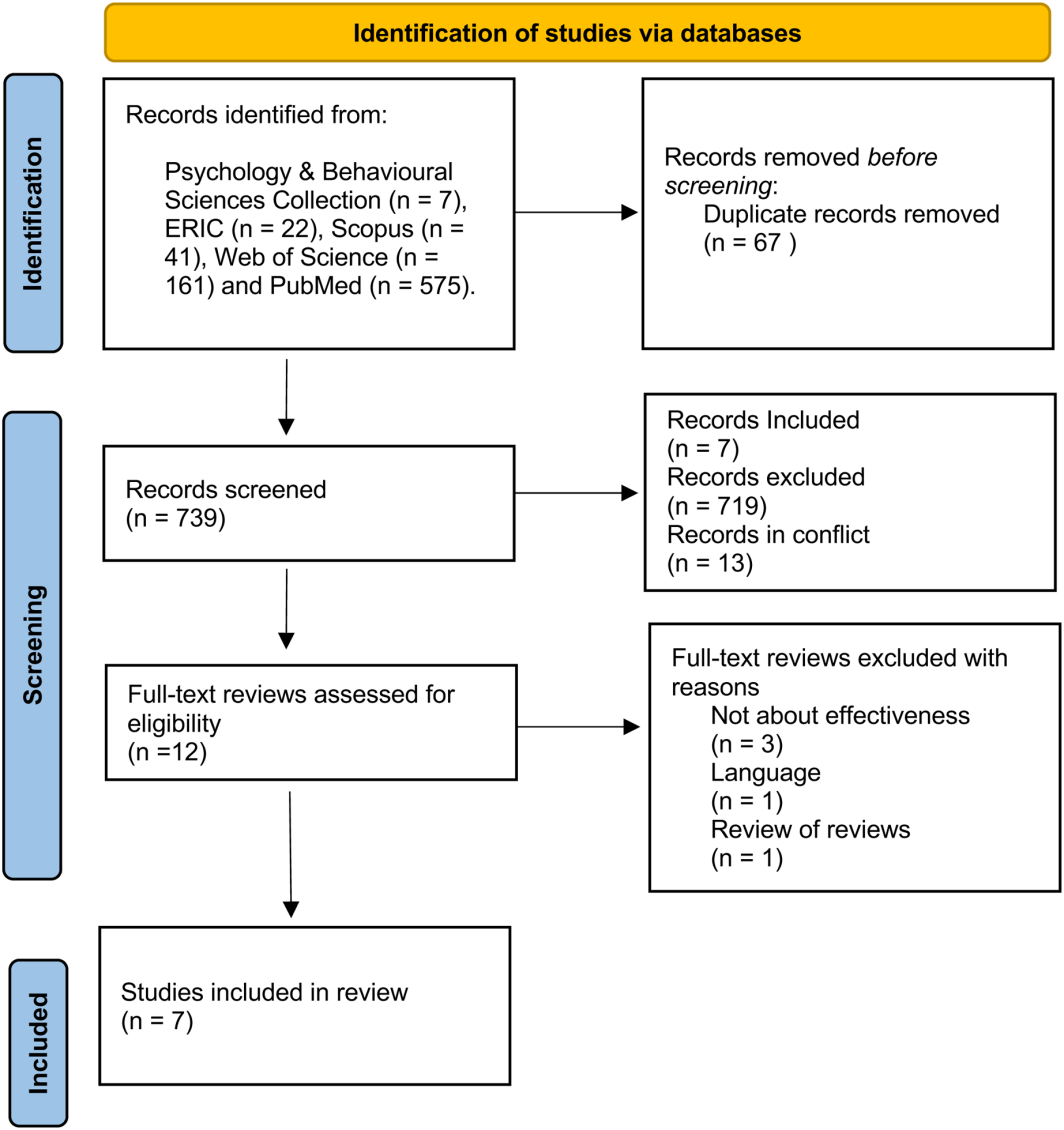
PRISMA flowchart.

The literature search resulted in 806 records of which 739 remained after removing duplicates. Initial screening of title and abstracts excluded another 727 citations and the remaining (*n* = 12) were included for full-text evaluation. Of these 12 citations, a total of seven met the inclusion criteria and were selected for this review. The included reviews were either systematic reviews (*n* = 3), meta-analysis (*n* = 2) or both (*n* = 2). Figure 1 shows the PRISMA flowchart of the selection process. A list of excluded studies and reasons for exclusion can be found in Supplemental Material S2.

### Characteristics of included reviews

The characteristics of the included reviews are shown in Table 1. The reviews analysed various primary studies of RCT (Bolinski et al., 2020; Conley et al., 2016; Davies, Morriss & Glazebrook, 2014; Farrer et al., 2013; Harrer et al., 2019), randomized (Conley et al., 2016; Farrer et al., 2013; Lattie et al., 2019), non-randomized (Lattie et al., 2019) and quasi-experimental design (Conley et al., 2016). The majority of reviews focused on undergraduates and postgraduates of 4-year colleges, graduate schools and various degree courses. Given the great heterogeneity of the studies, we did not conduct a statistical analysis of the data but described the results narratively.

**Table 1:** Characteristics of included reviews.

First Author, Year	Review type	Study design	No. of primary studies	Country of primary studies (*n*)	Intervention	Intervention target purpose group (*)	Targeted mental health issue	Effectiveness	Associated factors	Risk of Bias of primary studies	AMSTAR2 Score
Bolinski, 2020	Meta-Analysis	RCT	6	Australia, UK, USA (*n* = not reported)	CBT, Acceptance and commitment therapy, online expressive writing, web-based enhance meaning of life	Unspecified	Mood, Anxiety, Substance Misuse (Alcohol & Smoking)	Interventions had a positive effect for depression (g = −0.24 (95% CI [−0.46 to −0.03]; *p* = 0.03) and anxiety (g = −0.2 (95% CI [−0.3 to−0.09]; *p* ≤ 0.01).	Not reported	High	Moderate Quality
Conley et al., 2016	Meta-Analysis	Published/Unpublished reports, Randomised, Quasi-experimental control	48	USA (31) & all other countries (17)	CBT, Mindfulness, Psychoeducation, Social Skills, Relaxation, Online support groups, Others	Universal, Indicated	Depression; Anxiety; Stress; General psychological distress, Health, Social and emotional skills, self- perceptions, interpersonal relationships, spiritual outcomes	Universal interventions are effective for higher education students. Indicated interventions were reported significantly more effective (ES = 0.37) than universal interventions (ES = 0.19), partly due to higher symptom level students that participated	Program prevention level, Adherence	Not reported	Critically Low
Davies, Morriss & Glazebrook, 2014	Systematic Review & Meta-Analysis	RCT	17	Canada (3), Norway (1), Spain (1), UK (2), USA (7)	CBT, Mindfulness, Stress management theory and cognitive learning theory, lucid dreaming	Selective or Indicated, Treatment, Universal	Depression, Anxiety, Stress, Psychological distress, Social Anxiety, and Examination anxiety. General psychological well-being	Web-based and computer-delivered interventions can be effective in improving students’ depression (SMD −0.43; 95% CI [−0.63 to −0.22], *P* < 0.001), anxiety (SMD = −0.56; 95% CI [−0.77 to −0.35], *P* < 0.001), and stress outcomes (SMD −0.73; 95% CI [−1.27 to −0.19], *P* = 0.008) when compared to inactive controls	Adherence, User satisfaction	Moderate	Moderate Quality
Farrer et al., 2013	Systematic Review	RCT or Randomised	27	Australia (4), Belgium (1), China (1), Italy (4), Netherland (1), Spain (1), UK (2), USA (13)	CBT, Relationship skills training, Web-based social cognitive theory, Relaxation or Exposed based intervention, Stress inoculation training, CBT or education interventions, systematic desensitisation and relaxation, exposure therapy, virtual reality physical activity, health information & motivational feedback, hyponosis or biofeedback, writing, exposure expressive writing or auto-photic stimulation, education, motivational interviewing	Universal, Treatment, Selective, Indicated	Depression & Anxiety, Anxiety Symptoms, Examination anxiety, specific phobia, stress, social anxiety, computer-related anxiety, posttraumatic stress, generalised anxiety disorder, psychological distress, hardiness & acculturation, internet addiction	Out of 51 technology interventions, 47% associated with at least one positive outcome when compared to controls at post-test. With technology-based CBT as useful tool to target anxiety and depression	Not reported	Mean rating of 4.42 out of a possible 9	Critically Low
Harrer et al., 2019	Systematic Review & Meta-Analysis	RCT	48	Australia (5), Canada (3), Finland (1), Germany (2), Ireland (2), Norway (1), Romania (1), Spain (1), Sweden (1), UK (9), USA (22), Unknown (1)	CBT, Skills Training, Emotional Disclosure, personalised symptom and coping related feedback, bias modification procedures	Prevention, Treatment	Depression, Anxiety, Stress, disordered eating, well-being, and sleep.	Small effects on depression (*g* = 0.18, 95% CI [0.08 to 0.27]), anxiety *g* = 0.27, 95% CI [0.13–0.40]), and stress symptoms (*g* = 0.20, 95% CI [0.02–0.38]). Moderate effects on eating disorders symptoms (*g* = 0.52, 95% CI [0.22–0.83]) and role functioning (*g* = 0.41, 95% CI [0.26–0.56])	Recruitment, Available support, Adherence	23 studies (48%) reported low risk, 5 studies (10.2%), no studies met all criteria.	Moderate Quality
Lattie et al., 2019	Systematic Review	Randomised and Non-randomised	89	USA (46), UK (6), Ireland (5), Australia (5), Canada (5), China (5)	CBT & publicly available intervention	Universal, Treatment	Psychological well-being, psychological distress, Stress, Depressive, and/or anxious symptoms	Digital mental health interventions were either effective or partially effective in producing beneficial changes to the main psychological outcome	Adherence	28 studies were low risk, 9 studies were high risk, remaining 35 were judged as having some concern.	Moderate Quality
Rith-Najarian, Boustani & Chorpita, 2019	Systematic Review	Not reported	Of 62, 16 programs incorporated online/computer delivered	Not reported	Online/Computer delivered but unspecified	Universal, Selective, Indicated	Anxiety, Depression, and Stress. Stress only, Depression & Anxiety, Anxiety only, Anxiety & Stress, Depression only, Depression & Stress	Online/Computer delivered programs showed moderate effects	Program prevention level, Recruitment, Available support, Adherence	Not reported	Critically Low

**Notes:**

* Target Purpose Groups refer to; *Universal—*Interventions are available for all, and no screening is required, *Selected—*Interventions are for selected individuals that are at risk of a mental health condition, *Indicated—*Interventions are for those displaying symptoms of a given mental health condition.

RCT, Randomised Controlled Trials; CBT, Cognitive Behavioural Therapy; ES, Mean effect size.

### Quality assessment and risk of bias assessment

Using the AMSTAR-2 guidance to assess the quality of the manuscripts, three were classified as critically low (Conley et al., 2016; Farrer et al., 2013; Rith-Najarian, Boustani & Chorpita, 2019) and four were of moderate quality (Bolinski et al., 2020; Davies, Morriss & Glazebrook, 2014; Harrer et al., 2019; Lattie et al., 2019). AMSTAR-2 items most poorly reported include, mentioning the sources of funding of the primary studies (item 10) and stating an *a priori* establishment of methods prior to the review or registered protocol (item 2). Furthermore, most reviews (*n* = 6) listed general reasons for exclusion in their methods section but failed to report the specific reasons for excluding studies (item 7). Apart from that, all systematic reviews and meta-analyses used a comprehensive electronic literature search to identify potential articles, adequately discussed included studies, reported on heterogeneity, and any potential conflict of interest. The exact AMSTAR-2 judgement for each AMSTAR-2 domain can be found in Supplemental Material S3.

The quality ratings of the respective primary studies and the risk of bias assessment are reported in Table 1. The majority of the review authors used the Cochrane Collaboration risk of bias tool, and rated the primary studies as high (Bolinski et al., 2020), moderate (Davies, Morriss & Glazebrook, 2014; Lattie et al., 2019), and low (Harrer et al., 2019). One review (Farrer et al., 2013) used the Cochrane Effective Practice and Organization Care Group risk of bias criteria, which rated the primary studies a mean rating of 4.42 out of a possible 9. Two reviews did not assess the bias at all (Conley et al., 2016; Rith-Najarian, Boustani & Chorpita, 2019). Further, four reviews reported heterogeneity of included studies, of which three were high (Bolinski et al., 2020; Davies, Morriss & Glazebrook, 2014; Lattie et al., 2019), one moderate (Harrer et al., 2019), one low (Conley et al., 2016) and two were not reported (Farrer et al., 2013; Rith-Najarian, Boustani & Chorpita, 2019).

### Type of digital mental health interventions

The included reviews reported on a wide range of interventions delivered *via* digital platforms for various targeted purpose groups and mental health issues. Detailed information can be found in Table 1. The most reported type of intervention was internet-based Cognitive Behaviour Therapy (CBT) programs, which a total of six reviews investigated (Bolinski et al., 2020; Conley et al., 2016; Davies, Morriss & Glazebrook, 2014; Farrer et al., 2013; Harrer et al., 2019; Lattie et al., 2019). Other common interventions included forms of skills training (Conley et al., 2016; Harrer et al., 2019), mindfulness (Conley et al., 2016; Davies, Morriss & Glazebrook, 2014), and stress-related interventions (Davies, Morriss & Glazebrook, 2014; Farrer et al., 2013). The most common delivery format investigated by the reviews was internet-based, however also delivered across various digital platforms and devices such as emails, computerized programs, and virtual reality. Two reviews included mobile applications (Harrer et al., 2019, Lattie et al., 2019).

With regard to psychological outcomes, most systematic reviews and meta-analyses included in this umbrella review focused on depression (Conley et al., 2016; Davies, Morriss & Glazebrook, 2014; Farrer et al., 2013; Harrer et al., 2019; Lattie et al., 2019), anxiety (Bolinski et al., 2020; Conley et al., 2016; Davies, Morriss & Glazebrook, 2014; Farrer et al., 2013; Harrer et al., 2019; Lattie et al., 2019; Rith-Najarian, Boustani & Chorpita, 2019), and stress (Conley et al., 2016; Davies, Morriss & Glazebrook, 2014; Farrer et al., 2013; Harrer et al., 2019; Lattie et al., 2019; Rith-Najarian, Boustani & Chorpita, 2019). Other interventions focused on mood (Bolinski et al., 2020), substance misuse (Bolinski et al., 2020), psychological wellbeing (Davies, Morriss & Glazebrook, 2014; Harrer et al., 2019; Lattie et al., 2019), eating disorders (Harrer et al., 2019), and sleep (Harrer et al., 2019). Certain reviews included interventions for specific stress and anxiety problems such as psychological distress (Conley et al., 2016; Davies, Morriss & Glazebrook, 2014; Farrer et al., 2013; Lattie et al., 2019), post-traumatic disorder (Farrer et al., 2013), examination anxiety (Davies, Morriss & Glazebrook, 2014; Farrer et al., 2013), social anxiety (Davies, Morriss & Glazebrook, 2014; Farrer et al., 2013), computer-related anxiety (Farrer et al., 2013), and generalized anxiety disorder (Farrer et al., 2013).

The majority of reviews categorized the type of intervention based on the framework by Mrazek & Haggerty (1994) of targeted purpose groups, which are universal, selective, and indicated. Universal interventions are available for all, and no screening is required; selected interventions are for selected individuals who are at risk of a mental health condition, and indicated interventions are for those displaying symptoms of a given mental health condition. Based on these target groups, this would determine whether the intervention was for preventive or treatment purposes. Three reviews included all three intervention targeted purpose groups (Davies, Morriss & Glazebrook, 2014; Farrer et al., 2013; Rith-Najarian, Boustani & Chorpita, 2019), one review included universal and indicated interventions (Conley et al., 2016), one review included universal and treatment interventions (Lattie et al., 2019), one review classified as prevention and treatment interventions (Harrer et al., 2019) and one was unspecified (Bolinski et al., 2020).

### Effectiveness

The included reviews reported various digital mental health interventions. Due to great variation in reporting styles, we have categorised the interventions in two main ways; (a) type of intervention and (b) technology delivery method. To reiterate, reported effectiveness are interventions that were digitally delivered, and categories are not mutually exclusive. Overall, we found favourable evidence for the effectiveness of web-based online and computer delivered-interventions in reducing depression, anxiety, stress, and eating disorder symptoms (Davies, Morriss & Glazebrook, 2014; Harrer et al., 2019; Lattie et al., 2019, Rith-Najarian, Boustani & Chorpita, 2019; Bolinski et al., 2020). Similarly, positive results were found for skills-training interventions (Conley et al., 2016; Farrer et al., 2013) and CBT-based interventions (Davies, Morriss & Glazebrook, 2014; Farrer et al., 2013). The effectiveness for each delivery format is summarized below.

### Technology delivery method

#### Web-based, online/computer delivered-interventions

The review by Rith-Najarian, Boustani & Chorpita (2019) examined outcome-producing programs that targeted depression, anxiety, and/or stress and focused on interventions that were either group-based, self-administered, or delivered online. The effect sizes only slightly differed by delivery type such as the effect size were 0.69 (95% CI [0.58–0.81]) for group, 0.65 (95% CI [0.50–0.81]) for self-administered, and 0.52 (95% CI [0.41–0.63]) for online/computer-delivered interventions. However, analyses on the impact of publication bias on effect sizes did suggest the data is biased.

The review by Lattie et al. (2019) explored web-based intervention programs and found that the majority of digital health interventions were either effective or at least partially effective in producing beneficial changes in the psychological well-being of university students. Specifically, out of 71 studies that included web-based interventions, 30 were reported to be effective and 25 were partially effective. However, eight studies were not effective in improving psychological wellbeing and an additional eight, effectiveness analyses could not be performed. Due to the high heterogeneity of the data, a meta-analysis was not conducted.

The review by Bolinski et al. (2020) examined e-mental health interventions outcomes on depression and anxiety symptoms. The authors found that e-mental health interventions had a small and statistically significant effect on depression (*g* = −0.24, 95% CI [−0.46 to −0.03]) and anxiety (*g* = −0.2, 95% CI [−0.3 to −0.09]) when compared to inactive controls. Heterogeneity for this review was reported as high and results should be interpreted with caution.

The review by Harrer et al. (2019) explored the efficacy of internet interventions and found significant small-to-moderate effects on various mental health outcomes. More specifically, small significant effect were found for depression (*g* = 0.18, 95% CI [0.08–0.27]), anxiety (*g* = 0.27, 95% CI [0.13–0.40]), and stress (*g* = 0.20, 95% CI [0.02–0.38]). Further, significant moderate effects were detected for eating disorder symptoms (*g* = 0.52, 95% CI [0.22–0.83]). However, the authors noted heterogeneity was moderate to high for most of its analyses and after adjusting for publication bias, anxiety effects were not significant.

The review by Davies, Morriss & Glazebrook (2014) analyzed trials of web-based and computer-delivered interventions to mental wellbeing. Specifically, the authors analyzed seven trials on anxiety, nine trials on depression, two trials on psychological stress and three trials on general stress. The authors determined that the interventions can improve anxiety (pooled standardized mean difference [SMD] −0.56; 95% CI [−0.77 to −0.35]), depression (pooled SMD −0.43; 95% CI [−0.63 to −0.22]), and stress (pooled SMD −0.73; 95% CI [−1.27 to −0.19]).

Comparison interventions included a web-based stress management intervention, a face-to-face version of the intervention, another computer-based CBT program, and an online support group. Of the five trials, only four trials were extracted for analysis and all reported depression and anxiety outcomes, two of which were trials of MoodGym, a web-based CBT mobile application. For anxiety, neither interventions nor comparisons favored each other (*n = 198*, 4 RCTs, pooled SMD −0.10, CI [−0.39 to 0.18]). This was the same for depression outcomes as neither condition was favored (*n = 198*, 4 RCTs, pooled SMD 0.33, 95% CI [−0.43 to 1.09]). Heterogeneity for these analyses was reported as high.

#### Mobile applications and short message service

Lattie et al. (2019) included eight studies assessing the effectiveness of interventions delivered *via* mobile phones such as app-based programs and short message service-based programs. The authors found that most programs were effective in improving mental wellbeing.

#### Virtual reality interventions

Results concerning the effectiveness of virtual reality interventions were mixed. Lattie et al. (2019) emphasized that out of three studies investigating virtual reality programs targeting anxiety outcomes, one was effective and two were not.

The review by Farrer et al. (2013) analyzed the effectiveness of virtual reality interventions targeting anxiety symptoms, stress, and phobias. While the authors found that virtual reality interventions were not effective in reducing general anxiety symptoms and stress, positive outcomes were detected for spider phobia and acrophobia. Here virtual reality interventions were associated with significant reductions relative to a control group.

### Types of intervention

#### Computer-based or online delivered CBT

The review by Davies, Morriss & Glazebrook (2014) explored depression, anxiety and stress outcomes. More specifically, the authors analyzed these mental health outcomes in three subgroups comparing: (1) the intervention to an inactive control, (2) the intervention to active control, and (3) the intervention to another comparison intervention. The authors found that the web-based and computer-delivered interventions were only effective when compared to inactive controls (*i.e*., first subgroup), but not effective when compared to active controls (*i.e*., second subgroup) and comparison interventions (*i.e*. third subgroup). This finding was anticipated by the authors as they argue that participants in subgroup 2 and subgroup 3 were already actively involved in mental health counselling (*e.g*., face-to-face CBT).

The review by Farrer et al. (2013) explored technology-based intervention, in which approximately half of the included interventions were CBT. Mixed-effects were reported for depression and anxiety symptoms. Of the six CBT-based interventions, three were associated with a significant time × group interaction favoring the intervention group on both depression and anxiety symptoms outcomes. The remaining three CBT interventions only found effects for anxiety symptoms at post-intervention. Further, online CBT intervention was found to be effective for symptoms of examination anxiety and treating social anxiety disorder. The mean rating on the quality of studies was 4.42 out of 9.

#### Digitally delivered skills training

The review by Farrer et al. (2013) analyzed two relationship skills training interventions (RST) for their effectiveness on depression and anxiety symptoms. One RST had a significant interaction for depression symptoms at post-test, whilst another RST found anxiety symptoms after 10 months of follow-up.

The review by Conley et al. (2016) found that universal skill-training interventions were associated with a significant positive effect (effect size = 0.21, 95% CI [0.11–0.31]) while non-skill training programs were not effective (effect size = 0.15, 95% CI [−0.03 to 0.33]). However, these intervention types did not significantly differ from each other on a study level. Such universal skill-training interventions yielded significant effects for depression, anxiety, stress, and interpersonal relationships, whilst non-skilled training programs only showed significance for anxiety and health. Indicated skill-training interventions (effect size = 0.39, 95% CI [0.29–0.50]) and non-skill training programs were associated positive effects (effect size = 0.25, CI [0.01–0.49]) and did not differ from one another. Indicated skill training interventions yielded significant effects for depression, anxiety, stress, health, self-perception, interpersonal relationships. It also produced a significant effect on spirituality, but it was negative. On the other hand, indicated non-skilled programs only showed significant outcomes for depression and interpersonal relationships.

#### Digtially delivered relaxation and exposure-based therapy

Farrer et al. (2013) reported mixed effects for relaxation and exposure-based therapy. Two exposure-based interventions were effective for reducing anxiety relative to a control condition. Similarly, video and audio relaxation were associated with a significant within-group decline in anxiety symptoms although another study reported its ineffectiveness. Further, a study that examined exposure therapy and audio relaxation found computer-based delivery equivalent to group-based therapy. The mean quality rating for the study on examination anxiety was 3.67. Farrer et al. (2013) did note that one stress inoculation intervention did not provide sufficient data.

### Associated factors with effectiveness

Several of the included reviews had recognized factors that were associated with the effectiveness of interventions, including (a) program prevention level (Conley et al., 2016; Rith-Najarian, Boustani & Chorpita, 2019), (b) recruitment (Harrer et al., 2019; Rith-Najarian, Boustani & Chorpita, 2019), (c) available support (Conley et al., 2016), (d) adherence (Davies, Morriss & Glazebrook, 2014; Lattie et al., 2019; Harrer et al., 2019) and (e) user satisfaction (Davies, Morriss & Glazebrook, 2014). This review will provide a narrative analysis as follows.

#### Program Prevention Level

The review by Conley et al. (2016) found that the overall effectiveness of both universal and indicated interventions differed significantly from zero, with indicated interventions (effect size = 0.37, 95% CI [0.27–0.47]) yielding greater significant means effects than universal interventions (effect size = 0.19, 95% CI [0.11–0.28]). Effects at follow-up were significantly positive for both interventions (universal: effect size = 0.30, CI [0.06–0.54]; indicated: effect size = 0.49, CI [0.31–0.67]). The review by Rith-Najarian, Boustani & Chorpita (2019) noted that prevention programs were significantly more likely to be online/computer-delivered, which may have played a factor for the differing effect sizes at each prevention level (universal, *g* = 0.69; selective, *g* = 0.73; indicated, *g* = 0.53).

#### Recruitment

The review by Harrer et al. (2019) also noted that type of recruitment was a significant effect moderator for depression and stress outcomes, suggesting that effects were lowest when participants were recruited through a study subject pool (depression; *g* = 0.04, 95% CI [−0.10 to 0.17]; stress: *g* = −0.22, 95% CI [−0.70 to 0.27]). Effects were higher for web-based recruitment (depression: *g* = 0.30, 95% CI [0.25–0.57]; stress: *g* = 0.63, 95% CI [−0.05 to 1.31]). In addition, effects were higher in studies in which no compensation was provided (*g* = 0.31, 95% CI [0.18–0.45]) compared with studies that compensated participants (*g* = 0.08, 95% CI [−0.05 to 0.20]), although the effect size was not significant for studies with compensation. However, this differs from the review by Rith-Najarian, Boustani & Chorpita (2019) which found that the highest full completion rates were for studies with greater incentives.

#### Available support

The influence of available support remains mixed. The review by Conley et al. (2016) found that support available significantly moderated effects for indicated interventions yielding greater effects (effect size = 0.55, CI [0.37–0.72]) than self-administered indicated interventions (effect size = 0.28, CI [0.14–0.40]). However, such results differed from the findings of Harrer et al. (2019) review which suggested that support present during intervention did not affect intervention effectiveness (*p* ≥ 0.05).

#### Adherence

The review by Conley et al. (2016) suggested that how much of the intervention was completed by participants (*i.e*., dosage) moderated effects for 12 universal interventions. This showed that a higher percentage of intervention completers demonstrated better outcomes (*p* = 0.023). However, this was not the same for indicated interventions (*p* = 0.242).

The review by Davies, Morriss & Glazebrook (2014) noted that studies that adopted shorter interventions were associated with increased engagement and retention of participants. Further, one review showed that lower depressive symptoms were positively associated with increased adherence. However, in two trials there were no associations found between adherence and their level of post-intervention improvements on mental health.

The review by Lattie et al. (2019) found that studies that examined usability were generally favorable. However, many did report high rates of attrition and low rates of sustained program use. Although a direct comparison between studies cannot be conducted, a pattern emerged that module-based intervention usage dropped over an individual’s time spent in the study.

The review by Harrer et al. (2019) found that for interventions that focused on depression, effects were highest when interventions were between 4 and 8 weeks in duration (*g* = 0.31, 95% CI [0.13–0.49]) compared with shorter (*g* = 0.09, 95% CI [−0.02 to 0.21]) or longer (*g* = 0.13, 95% CI [−0.43 to 0.69]) programs.

#### User satisfaction

Davies, Morriss & Glazebrook (2014) found that studies administering participant evaluations to examine satisfaction were highly useable, satisfactory, credible, and were moderately-to-highly useful and helpful.

## Discussion

The present systematic umbrella review aimed to synthesize the literature on digital mental health interventions and to identify their effectiveness for improving psychological wellbeing among university students. Exploring seven systematic reviews and meta-analyses, we found evidence for the effectiveness of digital mental health interventions, particularly web-based online/computer delivered-interventions were effective or at least partially effective at decreasing depression, anxiety, stress, and eating disorder symptoms (Davies, Morriss & Glazebrook, 2014; Harrer et al., 2019; Lattie et al., 2019; Rith-Najarian, Boustani & Chorpita, 2019; Bolinski et al., 2020). Similarly, effectiveness was found for skills-training interventions (Conley et al., 2016; Farrer et al., 2013) and CBT-based interventions (Davies, Morriss & Glazebrook, 2014; Farrer et al., 2013). Although supported by only one review, mobile applications appeared to be effective in improving mental wellbeing (Lattie et al., 2019). For digital mental health interventions using virtual reality and relaxation, exposure-based therapy evidence was inconclusive (Farrer et al., 2013; Lattie et al., 2019). It is important to note that effectiveness was greatly dependent on the delivery format and mental health problems being studied. Furthermore, variations in study design, interventions, measures, and comparators, made it challenging to determine effectiveness accurately.

In addition, reviews also noted the presence of other influencing factors that were associated with intervention effectiveness. These include program prevention level, recruitment, available support, adherence, and user satisfaction. Differing effects have been found for prevention levels (Rith-Najarian, Boustani & Chorpita, 2019), with universal and indicated interventions producing an effect at post-intervention and follow-up (Conley et al., 2016).

Completion of intervention (*i.e*., dosage) influenced effectiveness, whereby participants that completed higher percentages of the intervention showed better outcomes (Conley et al., 2016). This could be affected by the duration of programs as shorter interventions were associated with better engagement and retention of participants (Davies, Morriss & Glazebrook, 2014; Harrer et al., 2019; Lattie et al., 2019). Available support provided during interventions and type of recruitment process may have contributed to intervention effectiveness, but this remains inconclusive due to mixed results (Harrer et al., 2019; Rith-Najarian, Boustani & Chorpita, 2019). Lastly, overall satisfaction of interventions may be associated with the effectiveness of interventions (Davies, Morriss & Glazebrook, 2014).

Despite the supporting evidence on digital mental health effectiveness, scholarly work within this area appears to be of moderate to critically low quality, according to the AMSTAR-2 assessment. This was due to not including a registered protocol or *a priori* and failure to report reasons for excluding studies and the source of funding. The included reviews also pointed out limitations such as substantial heterogeneity, varying sample sizes, intervention measures, and study and intervention design.

Overall, the findings of this umbrella review support previous findings on the beneficial effect of digital mental health interventions. Effectiveness has been determined amongst patients in Swedish clinical trials of internet-delivered CBT (Andersson, Carlbring & Rozental, 2019), veterans (Boykin et al., 2019), and older adults (Seifert, Reinwand & Schlomann, 2019). Reviews investigating digital mental health interventions amongst young people have found small effects in reducing depression and anxiety symptoms (Garrido et al., 2019), with specific effectiveness of computer-based CBT interventions (Lehtimaki et al., 2021). Nevertheless, within the context of young adults, who represent one of the most connected groups, digital mental health interventions are a promising tool and can help overcome structural and psychological barriers that university students may face when seeking mental health counselling. Moreover, students have reported that digital mental health services are convenient and easy to use (Hadler et al., 2021). Digital mental health interventions have helped to overcome issues of scheduling conflicts, waitlist, inaccessibility, and added expenses (Cohen, Graham & Lattie, 2020). Additionally, it allows individuals to avoid stigmatization of seeking mental health care by giving them a sense of ownership with their respective issues and facilitating help-seeking behaviours (Cohen, Graham & Lattie, 2020). However, evidence also points to concerns among students when it comes to digital mental health interventions. These include, for instance, concerns about privacy, developer credibility, and lack of guidance with digital mental health interventions. Acknowledging that user engagement could play a role in effectiveness, many studies have yet to assess this, which has been supported as future directions by other reviews (Bergin et al., 2020). Conceptualizing user experiences can enhance the personalization of digital mental health interventions, which has been shown to improve uptake, retention, and outcomes (Patel et al., 2020). Indeed, digital mental health interventions have been recognized to be attractive and relatable for younger populations (Garrido et al., 2019; Pretorius, Chambers & Coyle, 2019); however, challenges remain in determining adherence rates and attrition rates. This can be an important factor in determining intervention effectiveness, as reflected in our findings and others (Garrido et al., 2019; Becker & Torous, 2019). As suggested, digitally delivered interventions can improve participants engagement and retention amongst college students by providing opportunities for prompt reminders to ensure follow-up and completion (Hadler et al., 2021).

### Strength and limitations

Our review has several strengths and limitations that must be noted. The validity of our results depends on the quality of primary studies included in the systematic review and meta-analyses. According to our AMSTAR-2 assessment, the overall methodological quality of the review needs improvement. Certain AMSTAR-2 criteria were poorly addressed such as the prior protocol and list of excluded studies with the justification of exclusion. Hence, the overall confidence in the results of the systematic review and meta-analysis were of moderate or critically low quality. Thus, in line with most review authors, we emphasize the need to practice caution when interpreting the results as many had a high risk of bias. Similarly, review authors reported substantial heterogeneity. As previously reported in a review on internet- and computer-based interventions for depression, it has been suggested that eligibility criteria can play an important role as great variation in the symptomology of included participants may affect the overall power of the included interventions (Richards & Richardson, 2012). Additionally, the effectiveness of interventions was limited to accessible data. Review authors have noted the challenge in determining the sources of intervention effects such as elements that are responsible for driving larger effect sizes and moderating factors, and this is due to limited methods of assessments. For instance, only one review had reported on mobile applications, making it challenging to determine their true efficacy. Further, the variation in study settings, comparators, and inconsistencies in reporting, has proven to be a challenge for our umbrella review and others (Pretorius, Chambers & Coyle, 2019; Liverpool et al., 2020; Lehtimaki et al., 2021). Thus, this review calls for a more systematic approach to testing and reporting the effectiveness of digital mental health interventions.

Despite these limitations, the present umbrella review provides a detailed overview of the evidence of the beneficial effect of digital mental health interventions for university students. To the best of our knowledge, this umbrella review provides the first systematic synthesis of systematic reviews and meta-analyses on digital mental health interventions amongst university students. Prioritizing systematic reviews allowed us to synthesize a large amount of research evidence covering a wide variety of strategies, while at the same time narrowing it down to a particular interest group: university students. Nevertheless, our review has recognized a niche population with pressing issues and has brought light to research gaps within this research field to determine effectiveness holistically. This includes the need to assess adherence and completion rates amongst university samples by implementing separate and objective means to monitor usage as concluded to be an important issue in determining the efficacy of digital mental health support (Melville, Casey & Kavanagh, 2010). This can provide supporting evidence on the effectiveness and enhance understanding of program uptake and adoption specific to university students and campus communities. Furthermore, scholars should continue to examine and identify moderating effects for effectiveness, as suggested by our findings. Additionally, investigating digital mental health interventions within low-middle income countries (LMIC) would be important as students from LMICs are vulnerable to stressors that differ from the general population and those from higher-income countries. This includes limited access to healthcare services, poor diagnostic and treatment-seeking practices, and public and self-stigma associated with it. Thus, putting them in greater risk of mental health problems (Akhtar et al., 2020; Evans-Lacko & Thornicroft, 2019). Despite geographical and socio-economic disparities that are associated with digital access, it is reported that at least 43% of people from LMICs use the internet (International Telecommunication Union, 2019). Hence, this can be a promising approach in dealing with the prevalence rates of mental health issues amongst low-resource settings and provide greater generalizability of digital mental health interventions and conceptualization of university students mental wellbeing.

## Conclusions

This review supports the potential of digital mental interventions in overcoming the pressing mental health problems present amongst university students who actively use the interventions. Digital mental health interventions could be an effective alternative in dealing with current challenges and barriers faced by university students when seeking assistance with their mental health problems. Future studies should investigate user engagement and retention rates amongst university students to ensure sustainable effects and appropriate implementation of interventions.

## Supplemental Information

10.7717/peerj.13111/supp-1 Supplemental Information 1 PRISMA checklist.

10.7717/peerj.13111/supp-2 Supplemental Information 2 Search Strategy.

10.7717/peerj.13111/supp-3 Supplemental Information 3 Reasons for Excluding Studies.

10.7717/peerj.13111/supp-4 Supplemental Information 4 AMSTAR2 Ratings for Included Reviews.

10.7717/peerj.13111/supp-5 Supplemental Information 5 Rationale.

## Additional Information and Declarations

### Data Availability

The following information was supplied regarding data availability: All data summarised in this umbrella review are available in Table 1 and the Supplemental Files.

## References

[ref-1] Akhtar P, Ma L, Waqas A, Naveed S, Li Y, Rahman A, Wang Y (2020). Prevalence of depression among university students in low and middle-income countries (LMICs): a systematic review and meta-analysis. Journal of Affective Disorders.

[ref-2] American College Health Association (2017). American College Health Association-National College Health Assessment II: Fall 2017 reference group executive summary.

[ref-3] Andersson G, Carlbring P, Rozental A (2019). Response and remission rates in internet-based cognitive behaviour therapy: an individual patient data meta-analysis. Frontiers in Psychiatry.

[ref-4] Aromataris E, Fernandez R, Godfrey C, Holly C, Khalil H, Tungpunkom P, Aromataris E, Munn Z (2020). Chapter 10: umbrella reviews. JBI Manual for Evidence Synthesis. JBI.

[ref-5] Auerbach RP, Mortier P, Bruffaerts R, Alonso J, Benjet C, Cuijpers P, Demyttenaere K, Ebert DD, Green JG, Hasking P, Murray E, Nock MK, Pinder-Amaker S, Sampson NA, Stein DJ, Vilagut G, Zaslavsky AM, Kessler RC, WHO WMH-ICS Collaborators (2018). WHO world mental health surveys international college student project: prevalence and distribution of mental disorders. Journal of Abnormal Psychology.

[ref-6] Backhaus I, Varela AR, Khoo S, Siefken K, Crozier A, Begotaraj E, Fischer F, Wiehn J, Lanning BA, Lin P-H, Jang S-N, Monteiro LZ, Al-Shamli A, La Torre G, Kawachi I (2020). Associations between social capital and depressive symptoms among college students in 12 countries: results of a cross-national study. Frontiers in Psychology.

[ref-7] Becker TD, Torous JB (2019). Recent developments in digital mental health interventions for college and university students. Current Treatment Options in Psychiatry.

[ref-8] Bergin AD, Vallejos EP, Davies EB, Daley D, Ford T, Harold G, Hetrick S, Kidner M, Long Y, Merry S, Morriss R, Sayal K, Sonuga-Barke E, Robinson J, Torous J, Hollis C (2020). Preventive digital mental health interventions for children and young people: a review of the design and reporting of research. NPJ Digital Medicine.

[ref-9] Boykin DM, Keegan F, Thompson KE, Voelkel E, Lindsay JA, Fletcher TL (2019). Video to home delivery of evidence-based psychotherapy to veterans with posttraumatic stress disorder. Frontiers in Psychiatry.

[ref-10] Burns JM, Davenport TA, Durkin LA, Luscombe GM, Hickie IB (2010). The internet as a setting for mental health service utilisation by young people. Medical Journal of Australia.

[ref-11] Bolinski F, Boumparis N, Kleiboer A, Cuijpers P, Ebert DD, Riper H (2020). The effect of e-mental health interventions on academic performance in university and college students: a meta-analysis of randomized controlled trials. Internet Interventions.

[ref-13] Cohen KA, Graham AK, Lattie EG (2020). Aligning students and counseling centers on student mental health needs and treatment resources. Journal of American College Health.

[ref-14] Conley CS, Durlak JA, Shapiro JB, Kirsch AC, Zahniser E (2016). A meta-analysis of the impact of universal and indicated preventive technology-delivered interventions for higher education students. Prevention Science: The Officiall Journal of the Society for Prevention Research.

[ref-15] Davies EB, Morriss R, Glazebrook C (2014). Computer-delivered and web-based interventions to improve depression, anxiety, and psychological well-being of university students: a systematic review and meta-analysis. Journal of Medical Internet Research.

[ref-16] Evans-Lacko S, Thornicroft G (2019). WHO world mental health surveys international college student initiative: implementation issues in low-and middle-income countries. International Journal of Methods in Psychiatric Research.

[ref-17] Farrer L, Gulliver A, Chan JK, Batterham PJ, Reynolds J, Calear A, Tait R, Bennett K, Griffiths KM (2013). Technology-based interventions for mental health in tertiary students: systematic review. Journal of Medical Internet Research.

[ref-18] Garrido S, Millington C, Cheers D, Boydell K, Schubert E, Meade T, Nguyen QV (2019). What works and what doesn’t work? A systematic review of digital mental health interventions for depression and anxiety in young people. Frontiers in Psychiatry.

[ref-19] Hadler NL, Bu P, Winkler A, Alexander AW (2021). College student perspectives of telemental health: a review of the recent literature. Current Psychiatry Reports.

[ref-20] Hamza CA, Ewing L, Heath NL, Goldstein AL (2021). When social isolation is nothing new: a longitudinal study on psychological distress during COVID-19 among university students with and without preexisting mental health concerns. Canadian Psychology/Psychologie Canadienne.

[ref-21] Harrer M, Adam SH, Baumeister H, Cuijpers P, Karyotaki E, Auerbach RP, Kessler RC, Bruffaerts R, Berking M, Ebert DD (2019). Internet interventions for mental health in university students: a systematic review and meta-analysis. International Journal of Methods in Psychiatric Research.

[ref-22] International Telecommunication Union (2019). Mobile cellular subscriptions; individuals using the Internet. The World Bank. https://data.worldbank.org/indicator/IT.CEL.SETS?end=2019.

[ref-23] Karyotaki E, Cuijpers P, Albor Y, Alonso J, Auerbach RP, Bantjes J, Bruffaerts R, Ebert DD, Hasking P, Kiekens G, Lee S, McLafferty M, Mak A, Mortier P, Sampson NA, Stein DJ, Vilagut G, Kessler RC (2020). Sources of stress and their associations with mental disorders among college students: results of the world health organization world mental health surveys international college student initiative. Frontiers in Psychology.

[ref-24] Lattie EG, Adkins EC, Winquist N, Stiles-Shields C, Wafford QE, Graham AK (2019). Digital mental health interventions for depression, anxiety, and enhancement of psychological wellbeing among college students: systematic review. Journal of Medical Internet Research.

[ref-25] Lehtimaki S, Martic J, Wahl B, Foster KT, Schwalbe N (2021). Evidence on digital mental health interventions for adolescents and young people: systematic overview. JMIR Mental Health.

[ref-26] Li Y, Zhao J, Ma Z, McReynolds LS, Lin D, Chen Z, Wang T, Wang D, Zhang Y, Zhang J, Fan F, Liu X (2021). Mental health among college students during the COVID-19 pandemic in China: a 2-wave longitudinal survey. Journal of Affective Disorders.

[ref-27] Liverpool S, Mota CP, Sales CMD, Čuš A, Carletto S, Hancheva C, Sousa S, Cerón SC, Moreno-Peral P, Pietrabissa G, Moltrecht B, Ulberg R, Ferreira N, Edbrooke-Childs J (2020). Engaging children and young people in digital mental health interventions: systematic review of modes of delivery, facilitators, and barriers. Journal of Medical Internet Research.

[ref-46] Melville KM, Casey LM, Kavanagh DJ (2010). Dropout from Internet-based treatment for psychological disorders. British Journal of Clinical Psychology.

[ref-28] Mrazek PJ, Haggerty RJ (1994). Description of five illustrative mental disorders.

[ref-29] Page MJ, McKenzie JE, Bossuyt PM, Boutron I, Hoffmann TC, Mulrow CD, Shamseer L, Tetzlaff JM, Akl EA, Brennan SE, Chou R, Glanville J, Grimshaw JM, Hróbjartsson A, Lalu MM, Li T, Loder EW, Mayo-Wilson E, McDonald S, McGuinness LA, Stewart LA, Thomas J, Tricco AC, Welch VA, Whiting P, Moher D (2021). The PRISMA, 2020 statement: an updated guideline for reporting systematic reviews. BMJ.

[ref-30] Pan X-F, Wen Y, Zhao Y, Hu J-M, Li S-Q, Zhang S-K, Li X-Y, Chang H, Xue Q-P, Zhao Z-M, Gu Y, Li C-C, Zhang Y-Q, Sun X-W, Yang C-X, Fu C (2016). Prevalence of depressive symptoms and its correlates among medical students in China: a national survey in 33 universities. Psychology, Health & Medicine.

[ref-31] Patel S, Akhtar A, Malins S, Wright N, Rowley E, Young E, Sampson S, Morriss R (2020). The acceptability and usability of digital health interventions for adults with depression, anxiety, and somatoform disorders: qualitative systematic review and meta-synthesis. Journal of Medical Internet Research.

[ref-32] Pretorius C, Chambers D, Coyle D (2019). Young people’s online help-seeking and mental health difficulties: systematic narrative review. Journal of Medical Internet Research.

[ref-33] Richards D, Richardson T (2012). Computer-based psychological treatments for depression: a systematic review and meta-analysis. Clinical Psychology Review.

[ref-34] Richardson T, Elliott P, Roberts R, Jansen M (2017). A longitudinal study of financial difficulties and mental health in a national sample of British undergraduate students. Community Mental Health Journal.

[ref-35] Rith-Najarian LR, Boustani MM, Chorpita BF (2019). A systematic review of prevention programs targeting depression, anxiety, and stress in university students. Journal of Affective Disorders.

[ref-36] Seifert A, Reinwand DA, Schlomann A (2019). Designing and using digital mental health interventions for older adults: being aware of digital inequality. Frontiers in Psychiatry.

[ref-37] Shea BJ, Reeves BC, Wells G, Thuku M, Hamel C, Moran J, Moher D, Tugwell P, Welch V, Kristjansson E, Henry DA (2017). AMSTAR 2: a critical appraisal tool for systematic reviews that include randomised or non-randomised studies of healthcare interventions, or both. BMJ.

[ref-38] Slavich GM, Auerbach RP, Butcher JN, Hooley JM (2018). Stress and its sequelae: depression, suicide, inflammation, and physical illness. APA Handbook of Psychopathology: Understanding, Assessing and Treating Adult Mental Disorders.

[ref-39] Stellefson M, Hanik B, Chaney B, Chaney D, Tennant B, Chavarria EA (2011). eHealth literacy among college students: a systematic review with implications for eHealth education. Journal of Medical Internet Research.

[ref-40] Sussman S, Arnett JJ (2014). Emerging adulthood: developmental period facilitative of the addictions. Evaluation & the Health Professions.

[ref-41] Waqas A, Khan S, Sharif W, Khalid U, Ali A (2015). Association of academic stress with sleeping difficulties in medical students of a Pakistani medical school: a cross sectional survey. PeerJ.

[ref-42] Wathelet M, Duhem S, Vaiva G, Baubet T, Habran E, Veerapa E, Debien C, Molenda S, Horn M, Grandgenèvre P, Notredame C-E, D’Hondt F (2020). Factors associated with mental health disorders among university students in France confined during the COVID-19 pandemic. JAMA Network Open.

[ref-43] Winther D, Livingstone S, Saeed M (2019). Growing up in a connected world.

[ref-44] WHO (2016). Global diffusion of eHealth: making universal health coverage achievable: report of the third global survey on eHealth.

[ref-45] Xiao H, Carney DM, Youn SJ, Janis RA, Castonguay LG, Hayes JA, Locke BD (2017). Are we in crisis? National mental health and treatment trends in college counseling centers. Psychological Services.

