# Supplementary material for: Effectiveness of digital mental health interventions for university students: an umbrella review
Source: PeerJ. 2022 Mar 31;10:e13111. doi: 10.7717/peerj.13111 (PMC8977068; doi:10.7717/peerj.13111)
Supplement: Supplemental Information 2 [file peerj-10-13111-s002.docx]

| **Supplementary Material 1. Search strategy with pre-defined keywords** | |
| --- | --- |
| *Search Query* | *Keywords in title, abstract and keyword* |
| 1 | wellbeing OR "mental health" OR stress OR "psychological health" OR anxiety OR "mental illness" OR psychiatric OR depress* OR “mental disorder” OR “mood disorder” |
| 2 | online OR internet OR web OR digital OR computer OR eHealth OR “electronic health” OR mobile OR mHealth OR app OR apps OR application* OR phone OR smartphone |
| 3 | universit* OR college* OR "tertiary education" OR "higher learning" OR "higher education" OR "higher institution" |
| 4 | student* OR undergraduate* OR postgraduate* |
| 5 | Review OR “meta-analysis” |
| Final | 1+2+3+4+5 |
| Databases: PUBMED, Psychology and Behavioural Science Collection, Web of Science, ERIC, and Scopus  Publication Dates: January 1, 2000 to February 26, 2021. | |
