# Supplementary material for: Effectiveness of digital mental health interventions for university students: an umbrella review
Source: PeerJ. 2022 Mar 31;10:e13111. doi: 10.7717/peerj.13111 (PMC8977068; doi:10.7717/peerj.13111)
Supplement: Supplemental Information 3 [file peerj-10-13111-s003.docx]

| **Supplementary Material 2. Reasons for excluded studies** | | |
| --- | --- | --- |
| *First Author, Year* | *Review Title* | *Reason for exclusion* |
| Reavley et al., 2010 | Prevention and early intervention to improve mental health in higher education students: A review | Not a systematic review of primary evidence. |
| Montagni et al., 2020 | Mental Health-Related Digital Use by University Students: A Systematic Review | Did not discuss effectiveness of included interventions, primarily focused on barriers. |
| Pospos et al., 2018 | Web-Based Tools and Mobile Applications to Mitigate Burnout, Depression, and Suicidality Among Healthcare Students and Professionals: A Systematic Review | Did not discuss effectiveness of included interventions. |
| Saleh et al., 2017 | Stress management intervention via the Internet among students: Review of the literature | Not published in English. |
| Heesacker et al., 2020 | Computer‐assisted psychological assessment and psychotherapy for collegians. | Did not discuss effectiveness of included interventions, primarily focused on barriers. |
