# Supplementary material for: Effectiveness of digital mental health interventions for university students: an umbrella review
Source: PeerJ. 2022 Mar 31;10:e13111. doi: 10.7717/peerj.13111 (PMC8977068; doi:10.7717/peerj.13111)
Supplement: Supplemental Information 4 [file peerj-10-13111-s004.docx]

| **Supplementary Material 3. AMSTAR 2 ratings for included reviews** | | | | | | | | | | | | | | | | | |
| --- | --- | --- | --- | --- | --- | --- | --- | --- | --- | --- | --- | --- | --- | --- | --- | --- | --- |
| First Author, Year | Item 1 | Item 2 | Item 3 | Item 4 | Item 5 | Item 6 | Item 7 | Item 8 | Item 9 | Item 10 | Item 11 | Item 12 | Item 13 | Item 14 | Item 15 | Item 16 | Final Rating |
| Bolinski, 2020 | 1 | 0 | 1 | 1 | 1 | 1 | 0 | 1 | 1 | 0 | 0 | 1 | 1 | 1 | 1 | 1 | Moderate Quality |
| Conley, 2016 | 1 | 0 | 0 | 2 | 0 | 0 | 0 | 1 | 0 | 0 | 1 | 0 | 0 | 1 | 1 | 1 | Critically Low |
| Davies, 2014 | 1 | 0 | 1 | 1 | 0 | 0 | 1 | 1 | 1 | 0 | 1 | 1 | 1 | 1 | 1 | 1 | Moderate Quality |
| Farrer, 2013 | 1 | 0 | 0 | 2 | 1 | 1 | 0 | 1 | 1 | 0 | NA | NA | 0 | 1 | NA | 1 | Critically Low |
| Harrer, 2019 | 1 | 1 | 1 | 1 | 1 | 1 | 0 | 1 | 1 | 0 | 1 | 1 | 1 | 1 | 1 | 1 | Moderate Quality |
| Lattie, 2019 | 1 | 1 | 1 | 1 | 1 | 1 | 0 | 1 | 1 | 0 | NA | NA | 1 | 1 | NA | 1 | Moderate Quality |
| Rith-Najarian, 2019 | 1 | 0 | 1 | 0 | 0 | 0 | 0 | 1 | 0 | 0 | 0 | 0 | 0 | 0 | 0 | 1 | Critically Low |
| Items 2, 4, 7, 9, 11, 13, 15 are critical items that could critically affect the validity of the review and its final rating. 0 = No, 1 = Yes, 2 = Partial Yes, NA = Not applicable | | | | | | | | | | | | | | | | | |
