# Supplementary material for: Effectiveness of digital mental health interventions for university students: an umbrella review
Source: PeerJ. 2022 Mar 31;10:e13111. doi: 10.7717/peerj.13111 (PMC8977068; doi:10.7717/peerj.13111)
Supplement: Supplemental Information 5 [file peerj-10-13111-s005.docx]

1. The rationale for conducting the systematic review / meta-analysis;

Evidence on the effectiveness of digital mental health interventions, specifically amongst university students is a growing research field as argued to be an viable alternative to traditional forms of treatment. Further, it can be an appropriate method amongst university students as they represent a generation of digital natives with pressing mental health issues. Thus, it is important to synthesise the evidence and to inform of promising interventions in dealing with mental health disorders and enhancing psychological well-being.

1. The contribution that it makes to knowledge in light of previously published related reports, including other meta-analyses and systematic reviews

Preliminary searches show that growing amount of research focusing on identifying digital mental health efficacy for improving symptoms of various mental health problems and enhancing psychological wellbeing. However, to the authors’ knowledge, there are no previous umbrella reviews that specifically discuss digital mental health interventions for university students
